# Supplementary material for: The effects of genital myiasis on the diversity of the vaginal microbiota in female Bactrian camels
Source: BMC Vet Res. 2022 Mar 5;18:87. doi: 10.1186/s12917-022-03189-5 (PMC8897907; doi:10.1186/s12917-022-03189-5)
Supplement: Supplementary file 5 — Additional file 5. [file 12917_2022_3189_MOESM5_ESM.zip › MPL201709200_16s_yy/Treat1/css/js_css/jsPDF-master.zip!/jsPDF-master/docs/plugins_addhtml.js.html]

plugins/addhtml.js - Documentation


## Home

### Classes

- jsPDF

### Global

- addFont
- addHTML
- addMetadata
- addPage
- autoPrint
- CapJoinStyles
- circle
- ellipse
- getFontList
- lines
- lstext
- output
- rect
- roundedRect
- save
- setDisplayMode
- setDrawColor
- setFillColor
- setFont
- setFontSize
- setFontStyle
- setLineCap
- setLineJoin
- setLineWidth
- setPage
- setProperties
- setTextColor
- text
- triangle

# plugins/addhtml.js

```
/**
 * jsPDF addHTML PlugIn
 * Copyright (c) 2014 Diego Casorran
 *
 * Licensed under the MIT License.
 * http://opensource.org/licenses/mit-license
 */

(function (jsPDFAPI) {
	'use strict';

	/**
	 * Renders an HTML element to canvas object which added to the PDF
	 *
	 * This feature requires [html2canvas](https://github.com/niklasvh/html2canvas)
	 * or [rasterizeHTML](https://github.com/cburgmer/rasterizeHTML.js)
	 *
	 * @returns {jsPDF}
	 * @name addHTML
	 * @param element {Mixed} HTML Element, or anything supported by html2canvas.
	 * @param x {Number} starting X coordinate in jsPDF instance's declared units.
	 * @param y {Number} starting Y coordinate in jsPDF instance's declared units.
	 * @param options {Object} Additional options, check the code below.
	 * @param callback {Function} to call when the rendering has finished.
	 * NOTE: Every parameter is optional except 'element' and 'callback', in such
	 *       case the image is positioned at 0x0 covering the whole PDF document
	 *       size. Ie, to easily take screenshots of webpages saving them to PDF.
	 * @deprecated This is being replace with a vector-supporting API. See
	 * [this link](https://cdn.rawgit.com/MrRio/jsPDF/master/examples/html2pdf/showcase_supported_html.html)
	 */
	jsPDFAPI.addHTML = function (element, x, y, options, callback) {
		'use strict';

		if(typeof html2canvas === 'undefined' && typeof rasterizeHTML === 'undefined')
			throw new Error('You need either '
				+'https://github.com/niklasvh/html2canvas'
				+' or https://github.com/cburgmer/rasterizeHTML.js');

		if(typeof x !== 'number') {
			options = x;
			callback = y;
		}

		if(typeof options === 'function') {
			callback = options;
			options = null;
		}

		var I = this.internal, K = I.scaleFactor, W = I.pageSize.width, H = I.pageSize.height;

		options = options || {};
		options.onrendered = function(obj) {
			x = parseInt(x) || 0;
			y = parseInt(y) || 0;
			var dim = options.dim || {};
			var h = dim.h || 0;
			var w = dim.w || Math.min(W,obj.width/K) - x;

			var format = 'JPEG';
			if(options.format)
				format = options.format;

			if(obj.height > H && options.pagesplit) {
				var crop = function() {
					var cy = 0;
					while(1) {
						var canvas = document.createElement('canvas');
						canvas.width = Math.min(W*K,obj.width);
						canvas.height = Math.min(H*K,obj.height-cy);
						var ctx = canvas.getContext('2d');
						ctx.drawImage(obj,0,cy,obj.width,canvas.height,0,0,canvas.width,canvas.height);
						var args = [canvas, x,cy?0:y,canvas.width/K,canvas.height/K, format,null,'SLOW'];
						this.addImage.apply(this, args);
						cy += canvas.height;
						if(cy >= obj.height) break;
						this.addPage();
					}
					callback(w,cy,null,args);
				}.bind(this);
				if(obj.nodeName === 'CANVAS') {
					var img = new Image();
					img.onload = crop;
					img.src = obj.toDataURL("image/png");
					obj = img;
				} else {
					crop();
				}
			} else {
				var alias = Math.random().toString(35);
				var args = [obj, x,y,w,h, format,alias,'SLOW'];

				this.addImage.apply(this, args);

				callback(w,h,alias,args);
			}
		}.bind(this);

		if(typeof html2canvas !== 'undefined' && !options.rstz) {
			return html2canvas(element, options);
		}

		if(typeof rasterizeHTML !== 'undefined') {
			var meth = 'drawDocument';
			if(typeof element === 'string') {
				meth = /^http/.test(element) ? 'drawURL' : 'drawHTML';
			}
			options.width = options.width || (W*K);
			return rasterizeHTML[meth](element, void 0, options).then(function(r) {
				options.onrendered(r.image);
			}, function(e) {
				callback(null,e);
			});
		}

		return null;
	};
})(jsPDF.API);
```

  

Documentation generated by JSDoc 3.4.2 on Sun Oct 09 2016 11:08:27 GMT+0100 (BST) using the docdash theme.
